# The Effects of *Phyllanthus niruri Linn* on Infiltrating Dendritic Cell and Ratio of Neutrophile/Lymphocytes in Chemotherapy of Sprague-Dawley Rats with Colorectal Cancer

**DOI:** 10.31557/APJCP.2021.22.11.3561

**Published:** 2021-11

**Authors:** Michael Tendean, Ignatius Riwanto

**Affiliations:** 1 *Division of Digestive Surgery, Department of Surgery, Faculty of Medicine, Sam Ratulangi University / Prof. dr. R. D. Kandou Hospital, Manado, Indonesia.*; 2 *Department of Digestive Surgery, Faculty of Medicine Diponegoro University, Indonesia. *

**Keywords:** Phyllanthus niruri Linn, infiltrating dendritic cells, neutrophil/lymphocyte ratio, capecitabine

## Abstract

**Background:**

Chemotherapy as part of colorectal cancer management can cause death to immunologically active tumor cell, but also it has immune suppressive effect. Phyllanthus niruri Linn is known to has immunomodulatory effect. This study was intended to prove P. niruri Linn effect on infiltrating dendritic cells and Neutrophil/lymphocyte ratios (NLRs) in Sprague–Dawley rats with colorectal cancer which were given capecitabine chemotherapy.

**Methods:**

The study was randomized post–test only control group design. The samples were 39 Sprague–Dawley male rats, with body weight around 170–220 grams, induced by 1,2-dimetylhydrazine (DMH) 30 mg/kgBW once per week subcutaneously. On 9^th^,11^th^ and 13^th^ week, there were four induced rats sacrificed each week to detect colorectal cancer (CRC) development. On the 13th week, all of the 4 sacrificed rats developed colon cancer, so the induction had to be stopped. The rest of 27 induced rats were randomly divided into three groups: control-group (K) were left untreated (9 rats), group P1 (9 rats) were given Capecitabine and group P2 (9 rats) were given Capecitabine with combination of P. niruri Linn extract 13.5 mg/kgBW orally. After 17th week, all rats were terminated and tumor lesion of colon were processed to be paraffin blocks and were stained with HE for evaluating the NLRs, and immunohistochemistry (S100) for evaluating infiltrating dendritic cells. Data was analyzed by using Oneway-Anova-test and post-Hoc LSD-test. Considered significant if p was <0.05.

**Results:**

The mean±standard deviation of infiltrating dendritic cells showed increasing value in group P2 (62.11±31.35) compared to group P1 (52.78±29.24) though not statistically significant. The mean of NLRs also showed statistically significant elevation of value in group P2 (0.13±0.05) compared to group P1 (0.04±0.01).

**Conclusion:**

Extract of Phyllanthus niruri Linn increasing immunologic status through elevation of infiltrating dendritic cells and NLRs in animal model colorectal cancer with Capecitabine chemotherapy.

## Introduction

Colorectal cancer today is the third most commonly diagnosed malignancies and the fourth cause of death in the world, global burden is expected to be more than 60% with more than 2.2 million new cases and 1.1 million mortalities at the year of 2030 (Arnold et al., 2017). The incidence of colorectal cancer varies markedly worldwide. The incidence is high in countries with medium to high level of human development index such as Czech Republic and Slovakia in Eastern Europe, Japan in Asia, Australia, Western Europe and North America, but on the other hand, the colorectal cancer mortality rates in these countries are low. This is most likely the result of improvement on colorectal cancer screening and therapy management. (Bray et al., 2012; Center et al., 2009)

Although mortality of colorectal cancer is declining in the West (2.7 and 2.5% per year in men and women, respectively), death rates continue to rise in Asia. Report from WHO showed that the incidence of colorectal cancer is rapidly raising in many countries in Asia such as China, Japan, Korea and Singapore (Ng et al., 2013). According to GLOBOCAN 2020, colorectal cancer ranked sixth highest number of new cases and accounted for more than 9000 deaths cases in Indonesia (World Health Organization, 2020). In Dharmais Cancer Hospital, Indonesia 2013, colorectal cancer is the third most common malignancies after breast cancer and cervix cancer, with 269 new cases and mortality of 48 cases (Ministry of Health Indonesia, 2015).

The prognosis of colorectal cancer is principally based on the stage of the disease at the time of presentation and there is a significantly better survival outcome if detected in earlier stages. However, almost 33% of patients undergoing curative resection will relapse with recurrent disease secondary to unresected occult microscopic metastasis (Chang et al., 2006; Carethers, 2008).

The elimination of residual microscopic disease become the aim and target of adjuvant therapy, with the intention of curing and lowering the risk of cancer recurrence. It refers to treatment given in addition to, or following primary, surgical treatment. The systemic therapy of advanced colorectal cancer includes chemotherapy and biologic therapy, also known as targeted therapy, while radiotherapy is given to rectal cancer to lower local recurrence (National Cancer Management Committee, 2014; Brown et al., 2019). Despite improvement in surgical and adjuvant treatment, the survival outcome of patients with advanced colorectal cancer has still not provided satisfactory clinical result. Around 40% of colorectal cancer patients had recurrences during follow-up, and more than half of the patient died of cancer metastasis and the five-year survival rate is lower than 5%. This condition provides a chance for other therapeutic modality such as immunotherapy together to improve quality of life of colorectal cancer patient (Young et al., 2000; Sawitri et al., 2012). 

Immunotherapy has been a new approach in the multi-modality management of colorectal cancer, which aims to up-regulate the immune system in order to counter the process of carcinogenesis. There are three main categories of immunotherapy: monoclonal antibodies, immune response modifiers, and vaccines (Adam et al., 2003).

The compounds that can function as immune modulators are abundant in herbal plants. Phyllanthus niruri Linn, one of the herb species among the numerous amounts of medicinal plant which is widely known and used worldwide. It features multiple pharmacological properties such as immune modulator, anti-viral, antibacterial, diuretic, anti-hyperglycemia and hepatoprotector. Phyllanthus niruri Linn contains active phytochemicals in various part of it such as flavonoid, alkaloid, terpenoid, lignin, polyphenol, tannin, coumarin and saponin. Flavonoid and lignin are potential chemo – preventive agents in inhibiting cancer cell proliferation (Spelman et al., 2006; Oktaviati et al., 2012; Tjandrawinata et al., 2017). Immunotherapy could not be separated from the immune response of the host in tumor or cancer microenvironment. The tumor microenvironment is basically chronic inflammation due to the host immune response trying to suppress carcinogenesis.

Dendritic cells are professional antigen presenting cells, which function is to capture and present antigens to lymphoid organs which in turn will induce primary immune response from lymphocyte cells to suppress the cancer cells, in this particular case colorectal cancer cells. The infiltration of dendritic cells in primary tumor lesion is known for the significant survival improvement and the decreasing recurrences of different malignancies, colorectal cancer included (Dadabayev et al., 2004). Other than dendritic cells, part of the immune response also include the neutrophils and lymphocytes. The neutrophil / lymphocyte ratio (NLR) in other studies, is known to be the prognostic and predictive factor from different malignancies. In colorectal patients with metastasis limited to the liver, who underwent radical resection of the metastasis after neo-adjuvant chemotherapy, the high result of NLR predict worse prognosis (Dell’Aquila et al., 2018). One interesting thing is patients with lower NLR after neoadjuvant chemotherapy shows similar survival compared with patients whom NLR is low from the beginning for the 1st, 3rd, and 5th year survival (Kishi et al., 2009).

This study aims to evaluate the administration of Phyllanthus niruri Linn extract to 1,2 DMH induced Sprague – Dawley rats colon cancer with the usage of capecitabine as the chemotherapy management, using the infiltrating dendritic cells and neutrophil/ lymphocyte ratios (NLRs) as the parameter of immune response. Further researches are needed to optimize the treatment of advanced colorectal cancer especially combination chemotherapy as a part of systemic therapy and immunotherapy. This study was intended to prove the synergistic effect of chemotherapy and immunotherapy.

## Materials and Methods

The study was randomized “post–test only control group design”, and had been reviewed by the ethics commission board with the ethical clearance number 03/EC/H/FK-RSDK/I/2018. Samples were using 39 Sprague – Dawley rats with inclusion criteria: male, body weight are about 170–220 grams. The animal care and intervention were carried out according to the “Guide for the care and use of laboratory animals” and American Veterinary Medical Association (AVMA) animal welfare principles in Gadjah Mada University, Yogyakarta (Institute for Laboratory Animal Research, 2011). The whole process took time about 5 months.

The rats were injected by 1.2 DMH 30 mg/kgBW once per week subcutaneously. On 9th,11th and 13th week, there were four induced rats sacrificed each week to detect the development of colorectal cancer (CRC). On the 13th week, all of the 4 sacrificed rats developed colon cancer, so the induction had to be stopped. The rest of 27 induced rats were randomly divided into three groups: control group (K) was left untreated (9 rats), group P1 (9 rats) was given capecitabine and group P2 (9 rats) was given capecitabine with combination of Phyllanthus niruri Linn (PNL) extract 13.5 mg/kgBW orally. After 17th week, all rats were terminated and tumor lesion of colon were processed to be paraffin blocks and were stained with HE for evaluating the histopathology and the amounts of neutrophils and lymphocytes, in order to determine the neutrophil/ lymphocyte ratio (NLR), the paraffin blocks also stained with S100 as an immunohistochemistry examination to detect the infiltrating dendritic cells. Histopathology examination was conducted in Pathology Anatomy laboratory on medical faculty of Gadjah Mada University and dr. Sardjito General Hospital Yogyakarta. The data regarding variable dendritic cell and NLR were normally distributed, therefore it was analyzed by using Oneway Anova and LSD test, with significant consideration if p was <0.05.

## Results

**Figure 1:**
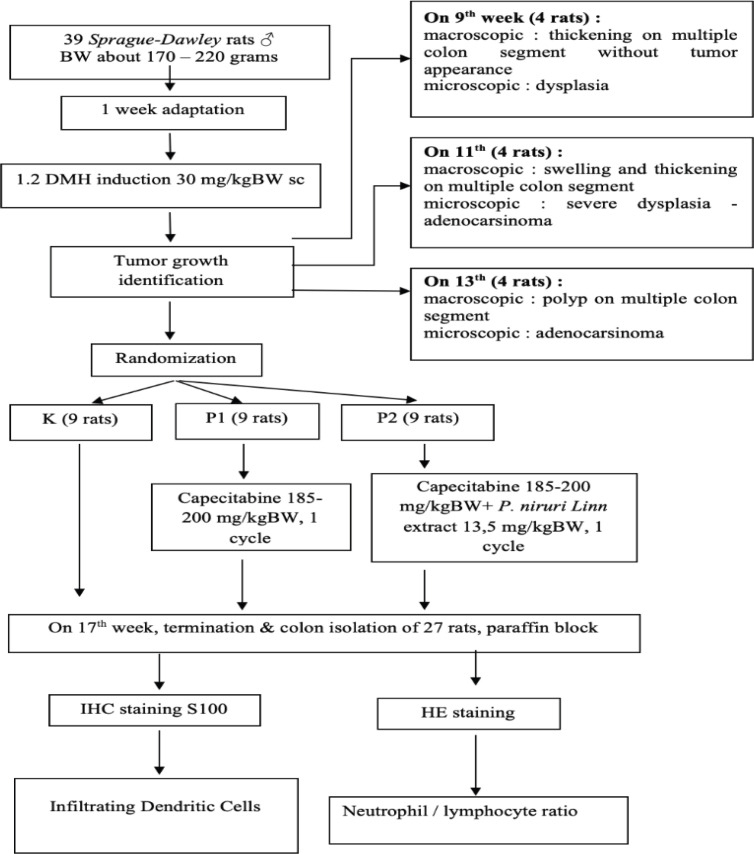
Consolidated Flowchart Report of Trial

All of the experimental animals were live and able to follow through the whole period of this study, from adaptation period to the 17th week, as can be seen in the consolidated report (Figure 1). All of rats were terminated at 17th week, after completed single cycle of chemotherapy with capecitabine, twice a day for 2 weeks and one week rest, and administration of immunotherapy with extract of Phyllanthus niruri Linn. Macroscopically, we observed the colon wall thickening and polyp in rat colon on each group. The tumor tissues were isolated and processed into paraffin block by Pathology Anatomy department. Each paraffin block was cut approximately 4 microns and stained with HE to confirmed its pathology. Adenocarcinoma colon was confirmed for all specimens; however, we did not distinguish the appearance of adenocarcinoma colon based on its location. Determination of group homogeneity among Sprague – Dawley rats in our study was performed with randomization and measurement of body weight on each group. The data distribution was tested using Kolmogorov-Smirnov test and the result is normal distribution. 

**Figure 2:**
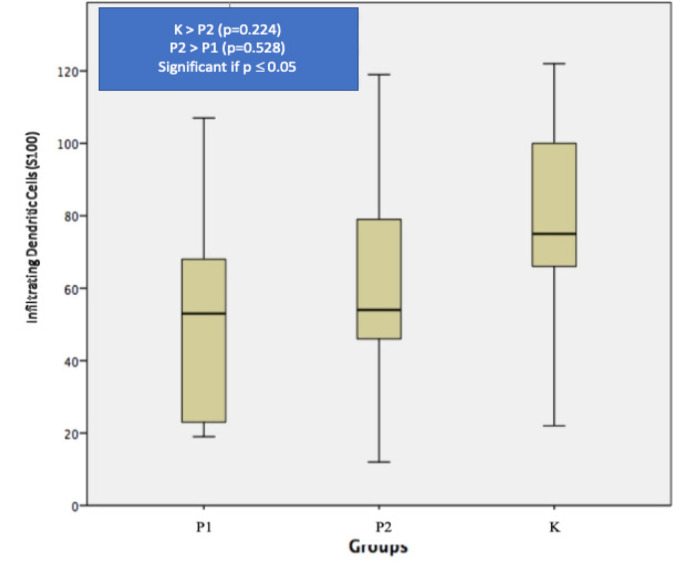
Box-Plot of Infiltrating Dendritic Cells Marked by S100, P1 = capecitabine group, P2 = capecitabine + *Phyllanthus niruri *Linn (PNL) group, K is control group

Analysis result of infiltrating dendritic cells on each group are as follows: mean of infiltrating dendritic cells on group K is 80.33 ± 32.15. While the mean of infiltrating dendritic cells on group P1 and P2 are 52.78 ± 29.24 and 62.11 ± 31.35 respectively. This comparison is depicted in figure 2. Normality test in our study uses Kolmogorov Smirnov and distribution of the data on all group are normal thus we continue hypothesis test with Oneway Anova and post-hoc analysis with LSD. The difference mean of infiltrating dendritic cells between three groups is statistically not significant (p > 0.05). Though not significant there are different mean of infiltrating dendritic cells in all three groups, with the highest being in K group, and the lowest amount being in P1 group, and the addition of Phyllantus Niruri Lynn in P2 group showing enhancement of infiltrating dendritic cells, which can be seen in Figure 2. 

**Figure 3:**
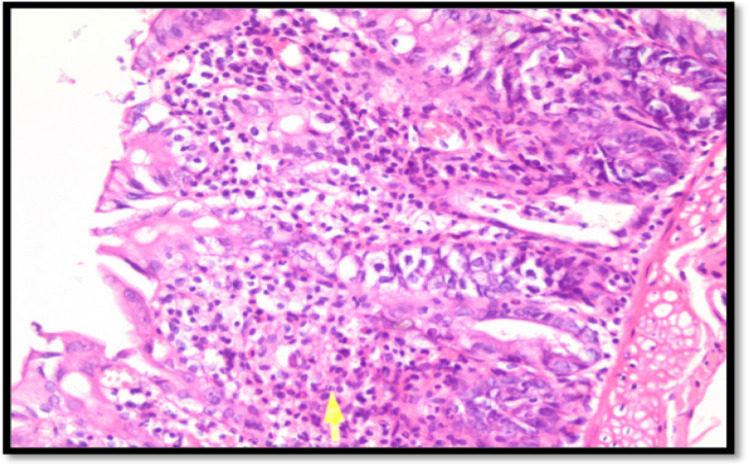
Hematoxylline Eosin (HE) Staining of Neutrophils, Marked with Yellow Arrow

**Figure 4:**
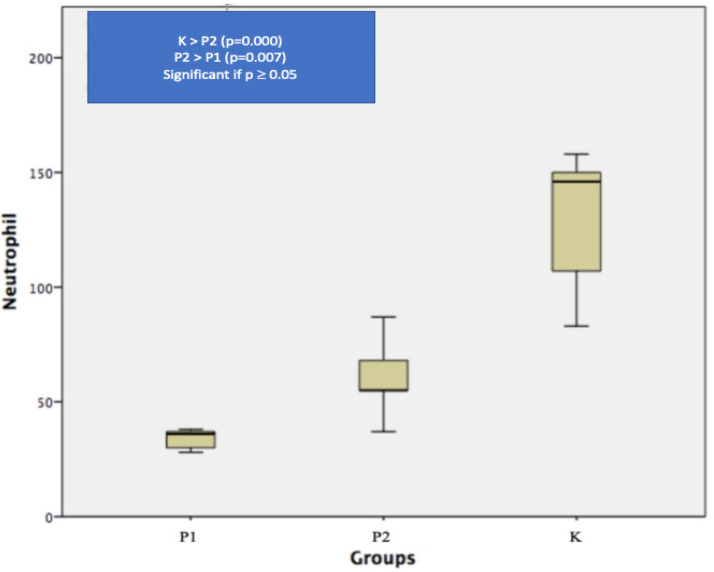
Box-Plot of Neutrophil Count, P1 = capecitabine group, P2 = capecitabine + Phyllanthus niruri Linn (PNL) group, K is control group

Infiltration of neutrophils to colon cancer tissue of Sprague – Dawley rats depict in the Figure 3. Analysis results to neutrophil count is as follows: the highest mean of neutrophil was found in control group (K) (132.56 ± 27.44), while P1 and P2 group has a mean of 34.22 ± 4.06 and 59.78 ± 15.43. Normalization test using Kolmogorov-Smirnov showed normal distribution in all three groups, thus we continue hypothesis test with Oneway Anova and post-hoc analysis with LSD. The mean differences of neutrophil count in all three groups is significant (p=0.000), which can be seen in Figure 4. 

**Figure 5:**
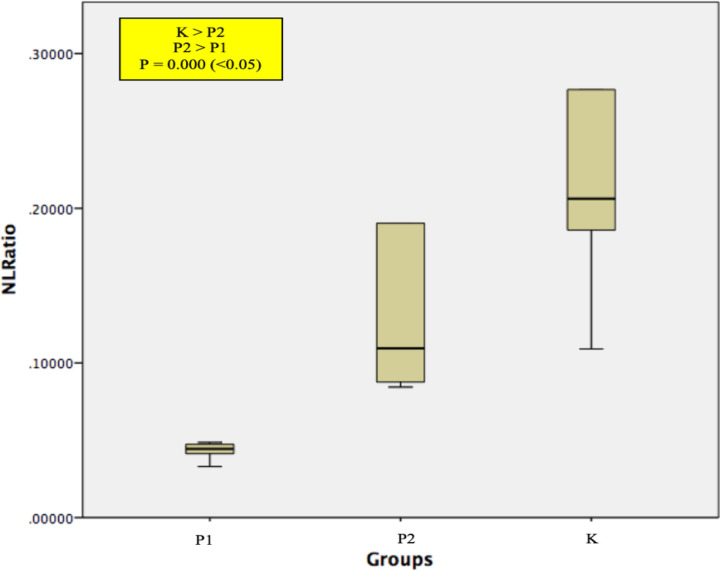
Box-Plot of neutrophil/ lymphocyte ratio, P1 = capecitabine group, P2 = capecitabine + *Phyllanthus niruri *Linn (PNL) group, K is control group

Analysis results to NLR can be seen in the Figure 5. The highest mean of NLR was in control Group (K) (0.21 ± 0.06), while group P1 (Capecitabine) and P2 (capecitabine and PNL) has a mean of value of 0.04 ± 0.01 and 0.13 ± 0.05. All groups have a normal data distribution, thus we continue hypothesis test with Oneway Anova and post-hoc analysis with LSD. The mean differences of NLR between all three groups were statistically significant (p= 0.000).

## Discussion

In our immune system, the body will activate the cellular and humoral immune response to counter against cancer cells. One of the immune system functions is to identify and eliminate cancer cells specifically, according to the specific tumor antigen expression. In order to identify specific tumor or cancer antigen, antigen presenting cells (APCs) are needed. As mentioned before, dendritic cells are professional APCs, which function is to capture, process, and present antigen to the surface of the cell layer together with other stimulator molecules. Tumor microenvironment is basically a chronic inflammation, and as all inflammation condition, dendritic cells will be attracted to the inflamed area, to capture and present antigen internally. Infiltration of dendritic cells in primary cancer cells has long been known to be related with significant survival improvement and the lower recurrence incidence in different kind of malignancies, including colorectal cancer (Dadabayev et al., 2004).

Ambe et al found that dendritic cells were detected with S100 immunohistochemistry with higher levels had significantly better 5 years survival compared to the lower levels of dendritic cells (70% vs 33%, P<0.001) (Ambe et al., 1998). Nakayama et al showed that significant lower levels of S100(+) dendritic cells are detected in colorectal cancer recurrences (p=0.003) (Nakayama et al, 2003). Gulubova et al showed that increasing S100(+) dendritic cells in cancer stroma had higher 5 years survival compared to the lower one (65% vs 35%, p=0.02) (Gulubova et al., 2012). This finding also supported by Nagorsen et al finding (60% vs 40%, p=0.04) (Nagorsen et al., 2007). We demonstrate that infiltrating dendritic cells levels is increased in the event of Phyllantus Niruri Lynn addition (62.11 ± 31.35), even though the highest level was found in control group (80.33 ± 32.15) in response to tumor growth due to 1.2 DMH induction.

Neutrophil/ lymphocyte ratio is well known to predict the outcome of patients and the response of therapy, a higher NLR correlate with worse prognosis and failure in response to therapy. Neutrophils as part of the immune system are the first responder to all inflammation condition, neutrophils will form a Neutrophil Extracellular Traps (NETs) which apparently are tumorigenic, by increasing cancer cells proliferation and inhibiting apoptosis (Powell et al., 2016). Increasing neutrophils in cancer microenvironment known as Tumor Associated Neutrophils (TANs), plays important rules in tumor progressivity, by releasing factors that modulate the extracellular matrix and inflammation of the tumor microenvironment of , such as neutrophil elastase (NE), neutrophil collagenase (MMP-8), and gelatinase B (MMP-9) (Shen et al., 2014). Besides pro inflammatory effect, neutrophils also suppress anti-cancer immunity, by releasing arginase-1 as an inhibitor of T cells function. This is shown in our study with the highest neutrophils count found in the control group (132.56 ± 27.44). As explained above, lymphocyte in tumor microenvironment plays important role in anti-cancer immunity. This study demonstrates higher NLR in control group, which relate to worse prognosis, this condition is in accordance with the facts that if chemotherapy as part of the management of colorectal cancer has not been applied whereas indicated, the prognosis is worse. 

In the clinical practice, chemotherapy is part of the colorectal cancer management. Five-fluorouracil (5-FU) is used in this study, due to its common usage as standard oral chemotherapy in colorectal cancer management. There are challenges in the usage of chemotherapy, due to its nature, chemotherapy targets all proliferating cells, not only cancer cells but also immune cells (chemotherapy induced cell death). 

Intensive usage of chemotherapy will in turn cause a large amount of reduction in lymphocytic population especially in B-cells and also ablation of T-cells function, this situation cause a decrease in anti-tumor response (Van der most et al., 2005; Ramarakrishnan et al., 2011; Bracci et al., 2014; Kanterman et al., 2014; Paschall et al., 2015). In this study we demonstrate, the P1 group given capecitabine chemotherapy shows “immunodepletion”, which can be seen from lower rate of infiltrating dendritic cells (52.78 ± 29.24) though not statistically significant, and statistically significant lower neutrophils (34.22 ± 4.06) and NLRs (0.04 ± 0.01) compared with the control group. 

Lymphodepletion due to chemotherapy effect has two unexpected benefits in modulating anti-tumor immune response. First, lymphodepletion causes amount decrease in CD4+ regulator T cells, which holds key role in keeping periphery tolerance and suppression of anti-tumor immune response, with the low amount or absence of these cells, the immunotherapy is able to induce T cells’ anti-tumor response. Second, lymphopenia triggers immune system regeneration phase, marked by lymphocyte proliferation homeostasis motored by interleukin (IL)-7 and IL-15, so the immune response could return to normal state in a period after chemotherapy (Kanterman et al., 2014; Paschall et al., 2015). 

As mentioned above, chemotherapy besides destroying cancer cells, also suppress immune system. This condition forced for an alternative more effective combination therapy, one of them including immunotherapy. Cancer immunotherapy has long been an interesting therapeutic approach for being low toxicity and high specificity (Powell et al., 2016). Immunotherapy works using certain substances as immunomodulator, these substances usually are contained in herbal medicine, one of them is Phyllanthus niruri Linn. It contains active phytochemical ingredients such as flavonoid, alkaloid, terpenoid, lignin, polyphenol, tannin, coumarin, and saponin (Tjandrawinata et al., 2017; Chen et al., 2015). Immune system improvement in colorectal cancer patients which receive capecitabine as part of the management, is demonstrated in this study by the increasing rate of infiltrating dendritic cells (62.11 ± 31.35 vs 52.78 ± 29.24) and statistically significant NLRs (0.13 ± 0.05 vs 0.04 ± 0.01) in Sprague – Dawley rats that receive capecitabine and Phyllanthus niruri Linn (P2) compared with capecitabine only (P1). 

The immunomodulator effect of PNL is also demonstrated by the significant raise of neutrophils count in P2 compared to P1 group (59.78 ± 15.43 vs 34.22 ± 4.06). The raise of infiltrating neutrophils in this group is due to number of apoptotic cells after the administration of 5-FU. There are three pathways to apoptosis from cells, which are intrinsic, extrinsic, and perforin-granzyme pathways related with Cytotoxic T-Lymphocyte cells and NK cells, after the apoptosis the following process is the phagocytosis of apoptotic cells. The rule of phagocytosis are performed by neutrophils, followed by macrophages and dendritic cells. This process also explained the elevated rate of the infiltrating dendritic cells in P2 group above (Elmore, 2007; Hart et al., 2008; Esmann et al., 2010).

These results are in coherent with the study conducted by Endang et al, 2013, that shows immune system improvement through the escalation of lymphocyte infiltration, perforin expression by CTL and NK cells, apoptosis index, the suppression of cell perforation and colorectal cancer growth on Sprague-Dawley rats induced with 1.2 DMH (Sawitri et al., 2013). In short, the use of Phyllanthus niruri Linn increases the expression of CTL and NK cells, in term also increases the amounts of apoptotic cells, which in respond to that is the phagocytosis process performed by neutrophils, macrophages, and infiltrating dendritic cells. The increasing amount of neutrophils as the phagocytosis response is a non-inflammatory one, the contact to or uptake of apoptotic cells inhibited neutrophil functions such as respiratory burst and the release of the proinflammatory cytokines TNF-α and interferon-inducible protein 10. In addition, because apoptotic cells inhibit proinflammatory functions of neutrophils, uptake of apoptotic cells by neutrophils contributes to the resolution of inflammation (Esmann et al., 2010).

Phytochemical substance in Phyllanthus niruri Linn such as flavonoid and lignin are chemopreventive agents which are potential in inhibiting cancer cells proliferation. Lignan initiates apoptosis in cell cycle by inhibiting telomerase activity, bcl2 suppression and activating c-myc, caspase 3 and caspase 8 to cancer cell lines HepG2, E1-1 monocyte, HeLa and MCP7; while flavonoid has been proven to inhibit proliferation and induce apoptosis to human colon cancer line Ca-co2, HT-29, and SW480 (Sawitri et al, 2013; Jose et al, 2014; Loo 2003; Giridharan et al, 2002).

As conclusion, Chemotherapy drug such as Capecitabine, can cause immune depletion by reducing infiltrating dendritic cells and the NLR. Extract of Phyllanthus niruri Linn has capability to compensate this effect when they are given together. The escalation of infiltrating dendritic cells and NLR are indicated after being given the extract of Phyllanthus niruri Linn to colorectal cancer Sprague – Dawley rats that were induced by 1,2 DMH. This proves that extract of Phyllanthus niruri Linn has a benefit as immunotherapy. Other study is still needed to find relationship between Phyllanthus niruri Linn and other chemotherapy agents as part of colorectal cancer management.

## Author Contribution Statement

Michael Tendean (MT) and Ignatius Riwanto (IR) designed the research protocol, conducting the research, discussing and writing the manuscript. 

## Ethical Statement

This research was approved by ethics commission board of Dr. Kariadi General Hospital with the ethical clearance number 03/EC/H/FK-RSDK/I/2018.

## Conflict of interest

The authors declare that there is no conflict of interest to declare 
